# IMG/VR v.2.0: an integrated data management and analysis system for cultivated and environmental viral genomes

**DOI:** 10.1093/nar/gky1127

**Published:** 2018-11-08

**Authors:** David Paez-Espino, Simon Roux, I-Min A Chen, Krishna Palaniappan, Anna Ratner, Ken Chu, Marcel Huntemann, T B K Reddy, Joan Carles Pons, Mercè Llabrés, Emiley A Eloe-Fadrosh, Natalia N Ivanova, Nikos C Kyrpides

**Affiliations:** 1 Department of Energy, Joint Genome Institute, Walnut Creek, CA, USA; 2 Biological Data Management and Technology Center, Lawrence Berkeley National Laboratory, 1 Cyclotron Road, Berkeley, CA, USA; 3 Department of Mathematics and Computer Science, University of the Balearic Islands, Spain

## Abstract

The Integrated Microbial Genome/Virus (IMG/VR) system v.2.0 (https://img.jgi.doe.gov/vr/) is the largest publicly available data management and analysis platform dedicated to viral genomics. Since the last report published in the 2016, NAR Database Issue, the data has tripled in size and currently contains genomes of 8389 cultivated reference viruses, 12 498 previously published curated prophages derived from cultivated microbial isolates, and 735 112 viral genomic fragments computationally predicted from assembled shotgun metagenomes. Nearly 60% of the viral genomes and genome fragments are clustered into 110 384 viral Operational Taxonomic Units (vOTUs) with two or more members. To improve data quality and predictions of host specificity, IMG/VR v.2.0 now separates prokaryotic and eukaryotic viruses, utilizes known prophage sequences to improve taxonomic assignments, and provides viral genome quality scores based on the estimated genome completeness. New features also include enhanced BLAST search capabilities for external queries. Finally, geographic map visualization to locate user-selected viral genomes or genome fragments has been implemented and download options have been extended. All of these features make IMG/VR v.2.0 a key resource for the study of viruses.

## INTRODUCTION

Viruses are ubiquitous, extremely abundant and diverse across all ecological niches (1). Traditional cultivation approaches remain challenging and have resulted in genomic characterization of only a small fraction of the virosphere (2). Advances in sequencing technologies and approaches to reconstruct uncultivated viral genomes directly from the environment have provided new avenues to catalog and explore the vast viral sequence space (3,4).

Various resources have been developed that host viral genomic data (5–7) or provide bioinformatics tools for viral data mining (8,9), although in many cases the metadata associated with available viral genomes (e.g. association with host(s), sampling location or sampled habitat) remains sparse or difficult to find. To fill this gap, we developed the Integrated Microbial Genome/Virus (IMG/VR) system, a data management and visualization platform providing multi-level integration of viral genomes and genome fragments, genes and gene clusters, protein functions and associated host and habitat data with analytical tools for comparative analysis of the global virome (10).

Here we present IMG/VR v.2.0 which now contains over 760,000 viral genomes and genome fragments from publicly available isolate genomes, curated prophages, and assembled metagenomes from the Integrated Microbial Genomes and Microbiomes (IMG/M) system (11). This update features refined viral operational taxonomic unit (vOTU) classification and genome quality scores based on estimated completeness that adhere to the recently proposed standards and best practices for describing uncultivated viral genomes (UViGs) (Roux *et al.*, in press, *Nature Biotech*). IMG/VR v.2.0 hosts an expanded scope of host-virus predictions through incorporation of similarity to known prophage sequences and domain-level assignment of viruses, and features new analytical tools, visualization options, and downloading capabilities.

## MATERIALS AND METHODS

### Domain-level host prediction

The 25,000 viral protein families (VPFs) used to identify UViGs were queried against the ViralZone database (12), where viral hosts were predicted at different taxonomic levels. 11 400 VPFs had at least one hit to the virus genomes and an average of 6.8 hits per model was calculated. For each VPF, a score value (between 0 and 1) was obtained dividing the total number of hits with a uniform distribution (only present in a single host domain) by the total number of VPF hits [i.e. score = (#uniform hits/#total hits)]. In the cases where the total number of hits was below the average number of hits, we corrected the score as follows: [(#uniform hits/#total hits) × (#total hits/average #hits)].

3788 VPFs were assigned with the maximum 1.0 score, representing those models found in at least seven known viral genomes and with a uniform domain distribution. The presence of these VPFs across the UViGs allowed us to separate 65% of the viral genomes into prokaryotic (bacteriophages and archaeal viruses), or eukaryotic viruses.

This approach has been benchmarked using the host assignment of the viral genomes containing pVOGs (13) with homology to our 1.0-score VPFs (2,037 pVOGs) with ≥95% homology based on hhsearch (14). Our classification was consistent with the classification in the pVOG database in all 98.6% of the cases. The remaining 1.4% resulted in viruses annotated as ‘archaea-bacteria’ viruses in the pVOG database that were identified as either bacteria or archaea using our approach. Thus, we can estimate that there was a 100% consistency of this method separating prokaryotic and eukaryotic viruses.

### Quality/Completeness of the UViGs

Following the recently developed ‘Minimum Information about an Uncultivated Virus Genome (MIUViG)’ framework, we classified the IMG/VR scaffolds as ‘genome fragment’ or ‘high-quality draft genome’ based on estimated genome completeness. The estimation of genome completeness relied on a predicted genome size calculated as follows.

First, circular contigs were identified based on identical sequences at 5′ and 3′ ends (≥10 bp), or repeats (direct or inverted) of 20 bp of more within 50 bp of the contig ends. To identify putative false positives, i.e. circular contigs that do not represent complete genomes, these circular contigs were affiliated first to ICTV genera (or families for ssDNA and RNA viruses) based on the presence of taxonomic marker VOGs (http://vogdb.org/) (Roux *et al.*, in press, *Nature Biotech*). VOG-based affiliations required ≥2 consistent VOG markers except for NCLDV where ≥5 markers were required, and for ssDNA and RNA viruses for which one marker was required. Alternatively, circular contigs were affiliated to genus-level groups including Viral RefSeq genomes using vContact2 (https://bitbucket.org/MAVERICLab/vcontact2) (15). For circular contigs grouped with isolated reference genomes, contigs with a length <80% of the minimum reference size of the group were considered as a false positive. For circular contigs in genus-level groups without any isolate, any circular contig with a length <80% of the maximum linear contig in the group was considered as a false positive. In addition, all circular contigs <10 kb and without any marker gene affiliation to a ssDNA or RNA virus genome were considered as false positives, as they likely originate from dsDNA virus genomes. Overall, 4166 circular contigs were considered as false-positive, and were analyzed as linear contigs for the purpose of completeness estimation (see below).

Linear contigs were first affiliated to ICTV genera based on the presence of ≥2 taxonomic marker VOGs (≥5 markers for NCLDV, ≥1 for ssDNA and RNA viruses), and their completeness estimated by comparison to the average genome length of isolates from the genus (or families for ssDNA and RNA viruses). Otherwise, linear contigs were affiliated to genus-level groups including Viral RefSeq genomes and circular contigs predicted as complete genomes (see above) using vContact2. For each genus-level group, an average genome length was first calculated based only on isolates and circular contigs. If the standard deviation of genome length in this group was ≤15% of the average genome length, this average was considered as the predicted genome size for all linear contigs in this group, as in (4). For linear contigs greater than the predicted genome size, the completeness was set at 99% as these are likely near-complete genomes.

## RESULTS

IMG/VR v.2.0 is the largest publicly available data management system for analysis and visualization of viral genomes and genome fragments integrated with associated metadata within the Integrated Microbial Genomes and Microbiomes system v.5.0 (IMG/M) (11).

### Data integrated into IMG/VR

#### Viral genomes and genome fragments

**Figure 1.**
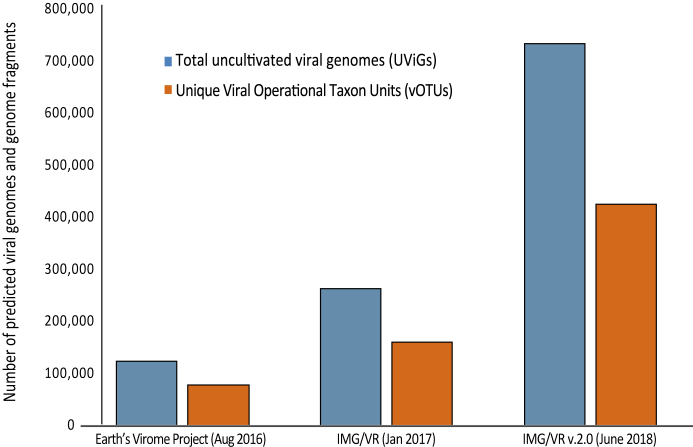
Growth rate of predicted viral genomes and genome fragments from publicly available assembled metagenomes. Growth during IMG/VR update cycles for total (UViGs) and unique (vOTUs) viral genomes and genome fragments from the Integrated Microbial Genome & Microbiomes (IMG/M) system using the JGI’s metagenomic virus discovery pipeline (16). Previous reports include the Earth's virome project (3) and the first release of IMG/VR (10).

The IMG/VR v.2.0 system contains 755 999 viral genomes and genome fragments from cultivated isolate virus genomes (iVGs) and uncultivated virus genomes (UViGs). This represents a 3-fold increase, as compared to the first public release in 2016 (3,10) (Figure 1) (3,16). The current 735 112 UViGs were identified from 7986 metagenomic samples integrated in the IMG/M system (11) from geographically and ecologically diverse habitats associated with the Genomes OnLine Database (GOLD) metadata and classification system (17). In addition, the data includes a total of 8389 reference viral genomes from the IMG/M database (11). These references represent high quality viral genomes with BioProject, BioSample and Assembly accession numbers from the NCBI virus database. Structural and functional annotation of all sequences is provided by the DOE Joint Genome Institute's annotation pipelines (18).

UViGs were identified using the JGI’s viral detection pipeline, as previously described (3,16). Briefly, a curated set of 25 000 viral protein families (VPFs) were used as bait to identify UViGs among assembled metagenomic sequences longer than 5kb followed by iterative filtering. The pipeline predictions are tuned to provide a highly specific detection of mostly lytic double-stranded DNA viruses and retroviruses (99.6% precision with a 37.5% recall rate) (16).

To complement iVGs and UViGs content, we incorporated a public set of 12 498 high-confidence viral genomes (prophages) detected in microbial host genomes (19). This dataset was used to create additional vOTUs (see below), and to enhance host-virus predictions helping to connect 3017 genome fragments with their specific hosts.

#### Viral OTU classification

All 755,999 viral sequences in IMG/VR (iVGs, UViGs, and prophages) were clustered into viral Operational Taxon Units (vOTUs), according to the recommendations of the new standards and best practices for describing genome sequences from uncultivated viruses (Roux *et al.*, in press, *Nature Biotech*). These vOTUs were generated via single linkage clustering of sequences with at least 95% average nucleotide identity (ANI; (20)) over at least 85% of the length of the shorter sequence.

In total, 442 675 sequences (representing 58% of the total) were clustered into 110,384 vOTUs (indicated with a ‘*vc*_’ prefix) ranging in size from 2 to 581 members per cluster. The majority of these vOTUs (51%) contain only two members, while ∼6% have 10 or more members. The remaining 317 778 sequences (42% of the total) were singletons (indicated with a ‘*sg*_’ prefix). Together, IMG/VR contains 428,162 vOTUs (including clusters and singletons) (Figure 1). Notably, the total number of newly predicted viral sequences and the number of vOTUs have grown linearly with the number of samples screened, indicating that viral sequence diversity estimates are not approaching saturation, as previously observed (3).

#### Viral-host specificity prediction

The prediction of putative host(s) for the UViGs has been split into two categories: specific host prediction and domain-level taxonomic assignment (prokaryotic and eukaryotic viruses).

Specific viral hosts were predicted using the computational approaches previously described (3). First, we propagated host assignments from the viral clusters containing iVGs (updated June 2018) and from the previously published curated prophage database (19), resulting in host prediction for 4212 UViGs from 671 viral clusters. Second, we used matches between viral sequences and the microbial CRISPR-Cas adaptive immune system, which hijacks small (from 25 to 65 base pairs) viral sequences and stores them within the microbial CRISPR arrays as spacers (21). This approach led to host prediction for 37 656 UViGs (including 4707 vOTUsand 7456 singletons). In total, we connected 49 bacterial and archaeal phyla and candidate phyla to viral sequences (Table 1). These complementary methods allowed us to discover previously unknown host-virus connections, including the identification of viruses predicted to infect hosts from 12 phyla for which no virus-host connections hitherto existed (Thermodesulphobacteria, Thaumarchaeota, Lentisphaerae, ca. Bathyarchaeota, ca. Micrarchaeota, ca. Desantisbacteria, ca. Aminicenantes, as well as candidate phyla within the CRP: ca. Wildermuthbacteria, ca. Moranbacteria, ca. Daviesbacteria, ca. Microgenomates and ca. Gracilibacteria),.

**Table 1.** Predicted bacterial and archaeal host phyla with corresponding number of UViGs. Archaeal phyla are indicated with (A)

Host phylum	Viral contig count
Euryarchaeota (A)	218
Crenarchaeota (A)	58
ca. Micrarchaeota (A)	40
Thaumarchaeota (A)	6
ca. Bathyarchaeota (A)	4
Aigarchaeota (A)	4
Nanoarchaeota (A)	2
Thermotogae (A)	1
Firmicutes	8123
Proteobacteria	5911
Bacteroidetes	3583
Actinobacteria	1971
Fusobacteria	1801
Spirochaetes	130
Verrucomicrobia	127
Synergistetes	58
Thermotogae	53
Chloroflexi	52
Cyanobacteria	47
Chlorobi	47
Deinococcus-Thermus	32
Aquificae	26
Fibrobacteres	21
Planctomycetes	15
Chlamydiae	14
Ignavibacteriae	12
Caldiserica	9
ca. Atribacteria	9
Gemmatimonadetes	8
ca. Desantisbacteria	7
Armatimonadetes	5
ca. Marinimicrobia	5
ca. Fervidibacteria	4
ca. Cloacimonetes	4
ca. Microgenomates	3
Marinimicrobia	3
ca. Moranbacteria	3
ca. Parcubacteria	3
ca. Aminicenantes	2
ca. Saccharibacteria	2
Nitrospirae	2
Tenericutes	2
ca. Wildermuthbacteria	2
ca. Daviesbacteria	2
ca. Omnitrophica	1
Lentisphaerae	1
Acidobacteria	1
ca. Gracilibacteria	1
Thermodesulfobacteria	1

*Host phyla without a previous connected virus. Microbial phyla classification according to the IMG/M system.

†Candidate Phyla from the CPR.

To perform domain-level host assignment of viral contigs, a new approach was developed that employs phylogenetic signature protein families and the presence of VPFs that are uniquely present in prokaryotic or eukaryotic viruses. Using this approach, domain-level host assignment was possible for 65% of the viral genomes and genome fragments. The vast majority of the IMG/VR v.2.0 data content (92%) are classified as prokaryotic viruses and reflects that the JGI viral detection pipeline targets mainly double-stranded DNA viruses and retroviruses.

### Availability of quality scores for viral genomes and genome fragments

**Figure 2.**
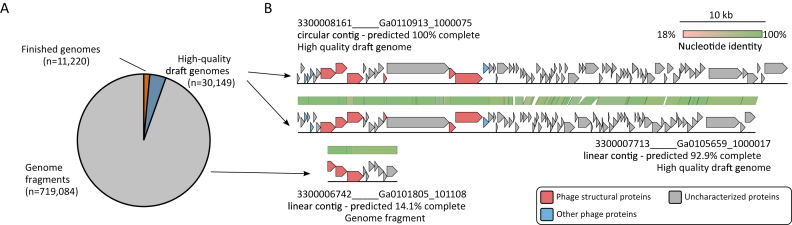
Distribution and example of the different viral genome quality categories in IMG/VR v.2.0. (**A**) Distribution of the number of sequences identified as ‘finished genome’, ‘high-quality draft genomes’, or ‘genome fragments.’ (**B**) Comparison of three contigs from vOTU_00079, two ‘high-quality draft genomes’ and one ‘genome fragment’. Genome quality category was based on estimated genome completeness (Roux *et al.*, in press, *Nature Biotech*). Genes are colored according to their functional annotation. The starting coordinate of the circular contig map was shifted to match the one of the linear contig maps.

Assembling viral genomes from metagenomes inevitably yields a heterogeneous mix of sequences ranging from short genome fragments to near-complete and even complete genomes. To help users separate complete and near-complete viral genomes from short fragments, we applied a genome completeness estimation method to classify viral sequences by genome quality according to community proposed standards (Roux *et al.*, in press, *Nature Biotech*). Briefly, circular contigs are considered putative complete genomes, with spurious matches removed based on their comparison to the closest isolate reference genome. Linear contigs are first clustered along with circular contigs and isolate reference genomes into genus-level groups. If the sizes of complete genomes from a genus-level group are consistent, the average size of these complete genomes is used as a ‘predicted genome size’ for all linear contigs in this group. In total, IMG/VR contains 11 220 finished isolate virus genomes (iVGs), with an additional 14 644 circular UViG contigs identified as likely complete genomes. A total of 15 505 linear contigs were classified as ‘high-quality draft genomes’ based on their estimated completeness of ≥90%, while the remaining 719 084 linear contigs were classified as ‘genome fragments’ due to their low completeness (*n* = 188 377) or lack of predicted genome size (*n* = 522 731) (Figure 2). Although this classification is based entirely on *in silico* predictions and would require *in vitro* experiments and/or isolation of the virus to ascertain genome completeness, this information is still valuable for users who want to focus their research on complete or near-complete predicted viral genomes.

### Data browsing and analysis features

The user interface of IMG/VR v.2.0 follows the same organizational principles as the original version (10). The IMG/VR homepage provides quick links to the different data categories (‘*Viral Datasets*’, ‘*vOTUs*’, viral genomes ‘*With Host*’ prediction, and viral genomes ‘*Quality*’ based on their genome completeness) in the upper left side, as well as ‘*Viral/Spacer BLAST*’ button (bottom left), and links for bulk download of the IMG/VR v.2.0 data content (Supplementary Figure S1). Users can also select viruses based on a specific geographic location (or human body sites) or based on the sample habitat type (upper and bottom right side, respectively) (Supplementary Figure S1).

**Figure 3.**
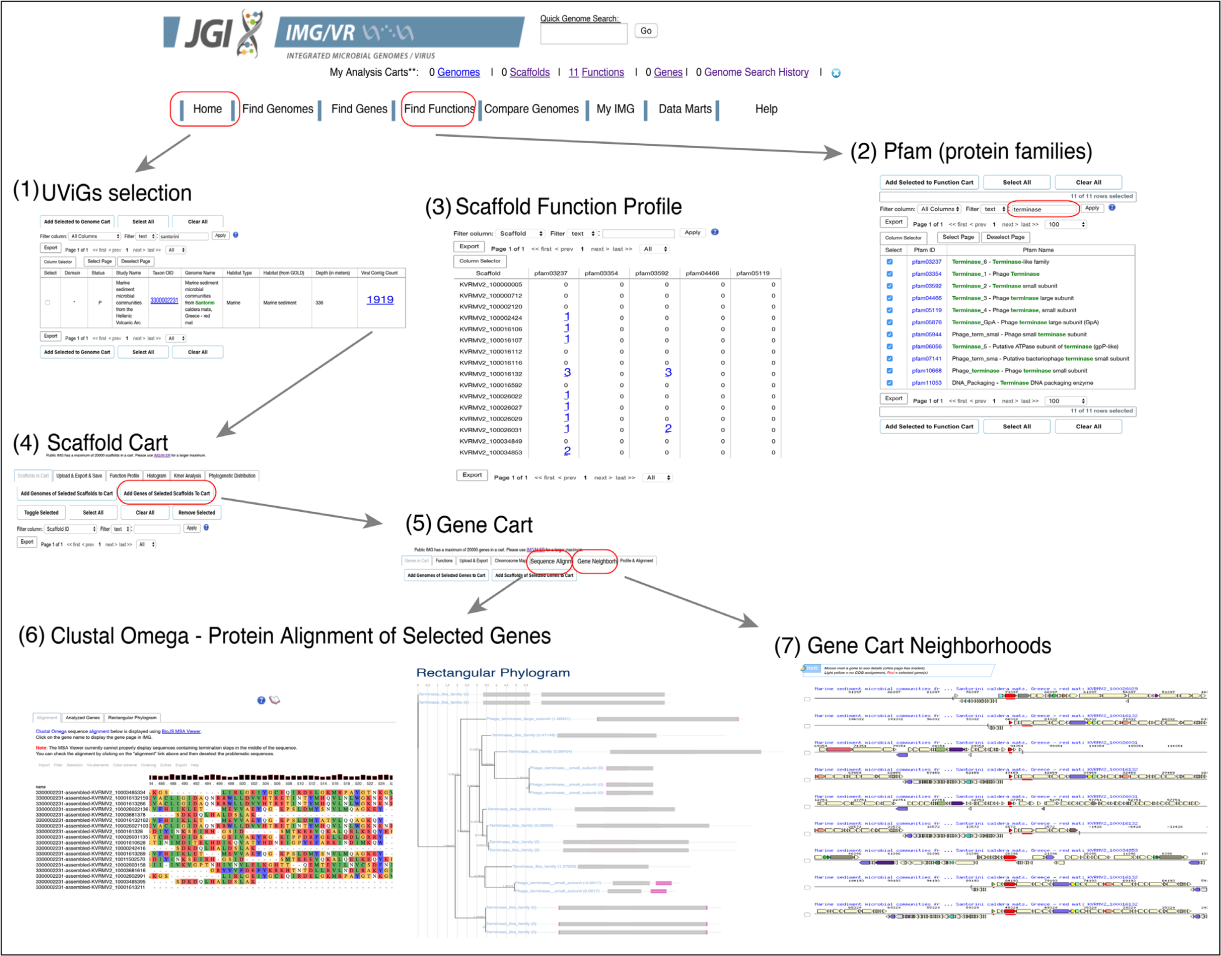
Example of analyses features in IMG/VR v.2.0. (1) UViGs were selected from a ‘*Viral Datasets*’ sample and added to the ‘*Scaffold Cart*’. (2) From the ‘*Find Functions*’ tab protein families (pfams) were filtered by the text ‘terminase’ obtaining pfams associated with this predicted viral function. (3) Located in the ‘*Scaffold Cart*’, the ‘*Scaffold Function Profile*’ option allows user to see the distribution of the selected functions (pfams in this example) against the selected list of UViGs. (4) Additionally, all genes from the selected UViGs can be added to the ‘*Gene Cart*’ by clicking ‘*Add Genes from Selected Scaffolds to Cart*’. (5) ‘*Gene Cart*’ functionality allows users to perform (6) gene or protein alignments of selected sequences and visualization as a phylogram or **(7)** to display the gene neighborhood of the selected genes (underscored in red). The location of the tools or the steps necessary to recreate this example are indicated in a red box.

Additionally, IMG/VR v.2.0 now offers *‘Scaffolds Cart’, ‘Genes Cart’* and *‘Functions Cart’* features analogous to those in the IMG/M system extending the collection and temporary storage of up to 20 000 entries for the duration of the session. These *Carts* include several tools that can be used for comparative genomics of UViGs, such as ‘*Scaffold Function Profile*’, ‘*Gene Function*’, ‘*Gene Chromosome Map*’, ‘*Gene Sequence Alignment*’ and ‘*Gene Neighborhoods*’, which enable users to visualize the synteny and functional content of viral sequences, as well as export the corresponding data of interest (Figure 3).

#### Scaffold function profile

This tab in ‘*Scaffold Cart*’ allows users to query a selected list of ‘*Functions’* including any that can be found under the ‘*Find Functions*’ menu added into the ‘*Function Cart*’ against UViGs of interest added to the ‘*Scaffold Cart*’. For example, a user can search all protein families associated with the function of ‘terminase’ to explore their presence and abundance across selected UViGs. Then, the genes encoding the selected function from the UViGs could be viewed by clicking on their counts.

#### Gene function

Genes from selected UViGs (e.g. those in the ‘*Scaffold Cart*’) can be added to the ‘*Gene Cart*’ (by using the ‘*Add Genes of Selected Scaffolds to Cart*’ button), and their association with predicted functions can be reviewed by using the *‘Functions’* button.

#### Gene chromosome map

From the ‘*Gene Cart*’, users can also visualize the genomic location of the selected genes by using the ‘*Chromosome Map*’ option.

#### Gene sequence alignment

Protein or DNA sequence alignments (using Clustal Omega (22)) of genes selected from the ‘*Gene Cart*’ can be quickly performed and visualized as rectangular phylograms by clicking the ‘*Sequence Alignment*’ button.

#### Gene neighborhoods

Chromosomal (UViG) neighborhood of genes selected from the ‘*Gene Cart*’ can be visualized (using selected directionality of the strands) by clicking on this tab.

All information from *‘Scaffolds’, ‘Genes’*, and *‘Functions’* tables can be independently accessed by clicking on their corresponding links or can be exported in a tab-delimited text format by using the *‘Export’* button. Phylogenetic trees can be exported in a Newick file format, and images as SVG or PNG files.

A new IMG/VR-ER (expert review) system (https://img.jgi.doe.gov/vr-er/), which will require a login/password access, will allow users to perform computationally intensive workspace-based analyses, analogous to the IMG/M-ER system (11).

### Google maps for geographic location of UViGs

**Figure 4.**
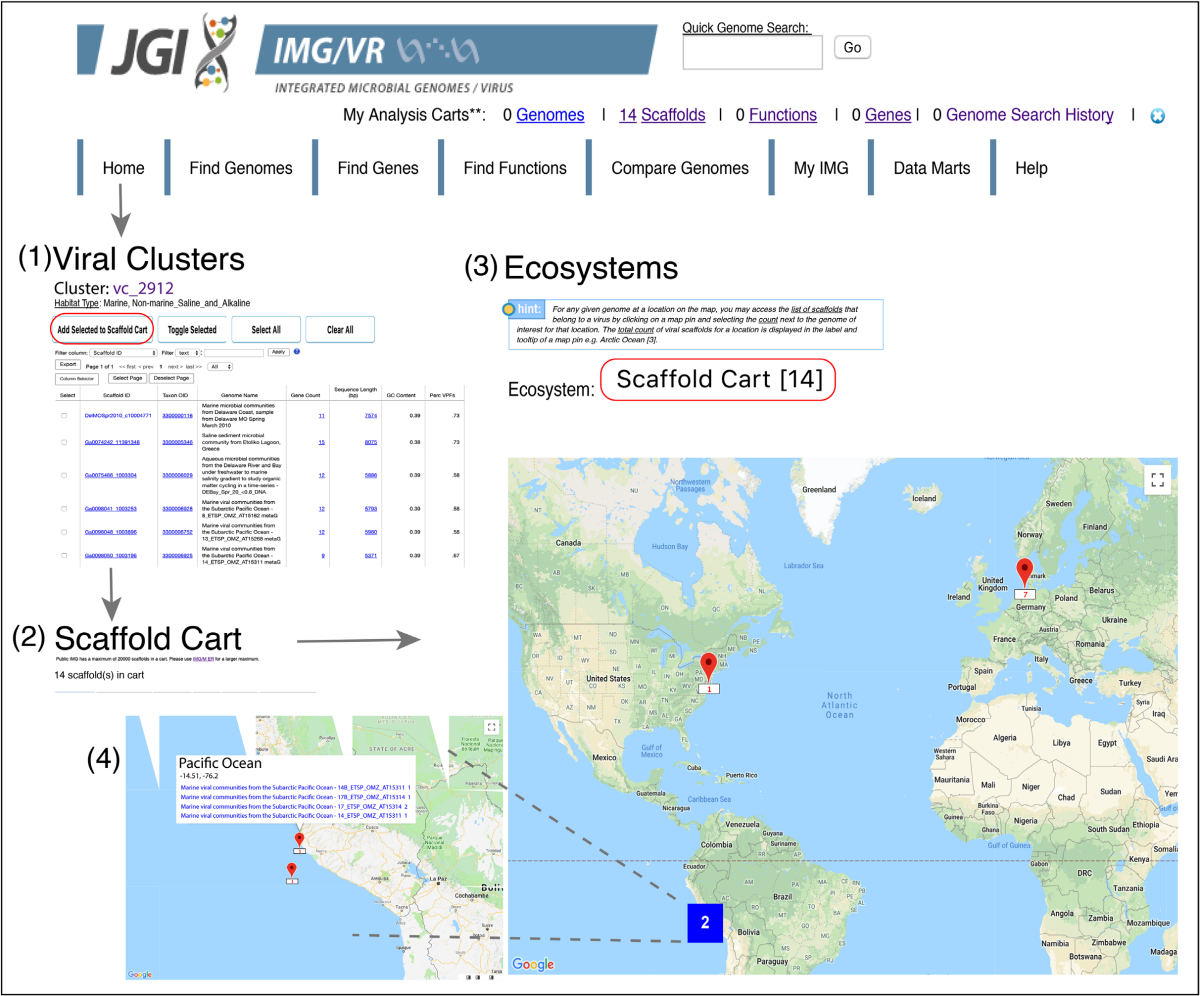
Visualization of geographic location from selected viral genomes. Uncultivated viral genomes and genome fragments (UViGs) can be accessed differently. (1) Here, UViGs from the viral cluster *‘vc_2912’* were selected from the ‘*Viral Clusters*’ link in the IMG/VR Home Page and added to the ‘*Scaffold Cart*’. (2) 14 UViGs were retrieved and a feature table displayed (not shown in the figure). (3) To obtain a Google Map with the location of the selected UViGs, users need to get back to the Home Page and select ‘*Scaffold Cart*’ from the ‘*Ecosystem*’ drop down box above the map. Map pins (in red) represent location counts of viral contigs and may contain multiple samples. Map pins are grouped into clusters (bold number in a coloured square based on number of members within the cluster) according to the Google Map javascript API utility library. (4) As you zoom into any of the cluster locations, the number on the cluster decreases, and you begin to see the individual markers on the map from which specific UViGs can be selected. Zooming out of the map consolidates the markers into clusters again.

UViGs of interest can be now selected into the ‘*Scaffold Cart*’ and visualized on a Google map. On the IMG/VR home page, a user can access this new functionality by selecting ’*Scaffold Cart*" from the ‘*Ecosystem*’ drop down box above the map. The ‘*Scaffold Cart*’ menu item only appears in the drop down if there are UViGs selected in the cart (Figure 4).

### Enhanced sequence similarity search features

**Figure 5.**
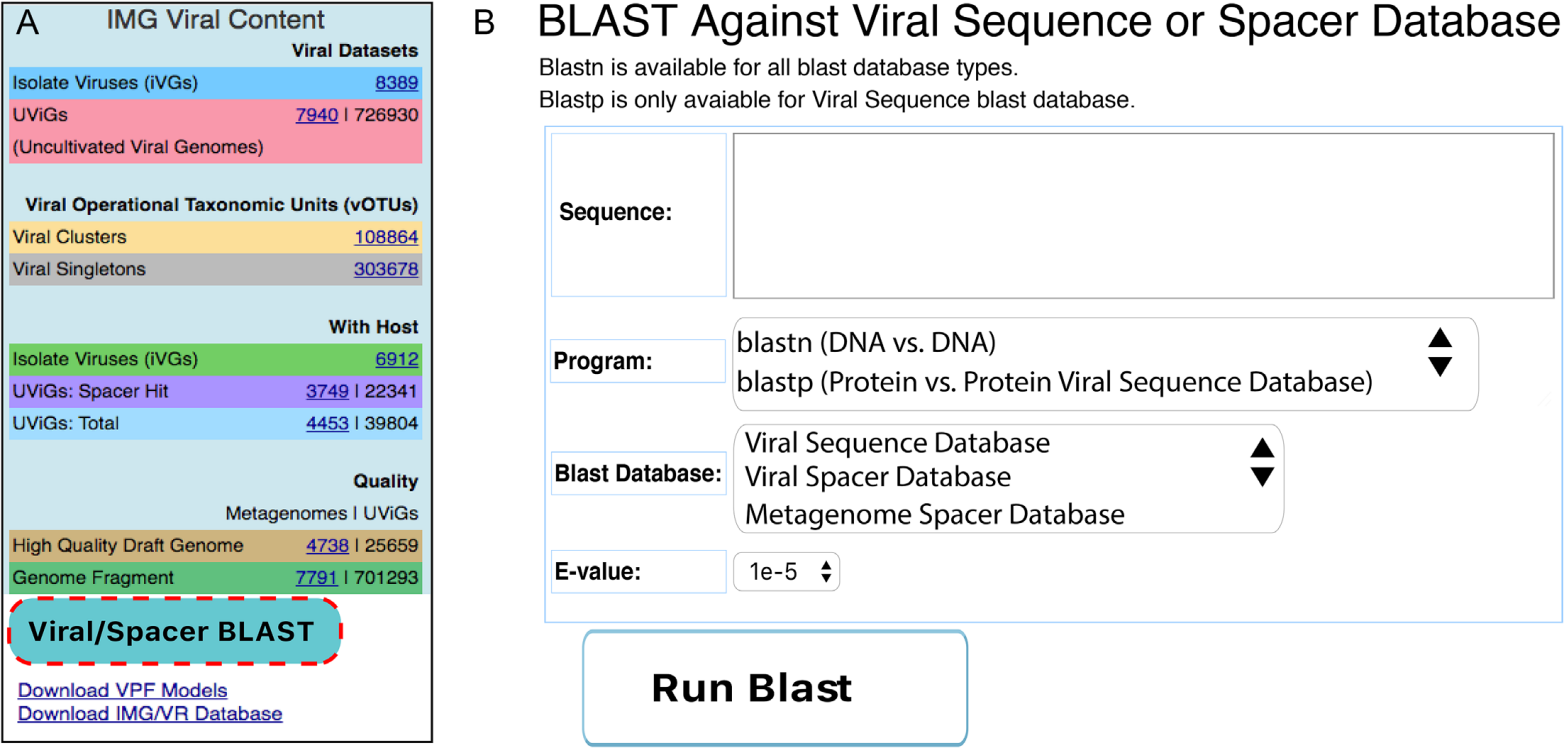
Searches against IMG/VR v.2.0 databases. (**A**) Location of the blast tool in IMG/VR v.2.0 (dashed red box). (**B**) User interface to blast sequences. Users can select between nucleotide or protein searches by selecting the blastn or blastp program when querying external ‘*Viral Sequences*’. For nucleotide searches, users can additionally use the displayed spacer ‘*Blast Databases*’.

Sequence similarity search options (bottom left of the home page under the ‘*Viral/Spacer Blast*’ button) have been extended in IMG/VR v.2.0 (Figure 5) to enable BLAST queries (23) of user-submitted sequences against the viral nucleotide or amino acid databases with customizable e-value cutoffs. Similarly, user-submitted nucleotide sequences can be queried using BLASTn against a database of predicted CRISPR spacer sequences derived from assembled metagenomes or isolate genomes in order to find matches to potential host(s) or habitat(s).

### Bulk download of IMG/VR v.2.0 data contents

In addition to the web interface which enables users to query and analyze viral genomes and genome fragments, IMG/VR v.2.0 now provides the bulk download options for all viral sequences, viral protein families (VPFs), and associated metadata. This allows users to perform additional analyses and large-scale computations using their own tools and computational resources. The viral sequence data can be downloaded through either the Joint Genome Institute's Genome Portal (https://genome.jgi.doe.gov/portal/IMG_VR/IMG_VR.home.html) under the ‘*Download*’ tab, or from the ‘*Download IMG/VR database*‘ button at the bottom left corner of the IMG/VR landing page (https://img.jgi.doe.gov/vr/) (Supplementary Figure S2). The files available to download include the nucleotide and amino acid sequences (in a FASTA format), as well as a table of extensive metadata including association of sequences with vOTUs, genome quality and completeness, predicted host specificity, as well as habitat information. Previous IMG/VR versions are also available for download on the same pages.

## DISCUSSION

The IMG/VR system was released two years ago as the largest publicly available viral-focused genomic repository with associated metadata and has been widely used in the research community (for example, (24–28)). Since the initial release, the IMG/VR content has tripled in size with over 755 000 viral genomes and genome fragments. Considering the number of newly identified UViGs and the growth of vOTUs, we project that the number of viral sequences hosted in IMG/VR will continue to grow superlinearly. We anticipate that this increase over time, however, will not affect the database infrastructure nor the system capabilities, since it only represents a small fraction of the original IMG/M total data. In this release, we have updated and added a number of features that expand the functionality of the resource. First, IMG/VR v.2.0 now adheres to community-accepted standards and best practices to characterize genome sequences from uncultivated viruses, including terminology (e.g. UViGs, vOTUs) and genome clustering methodology. Second, we have improved the virus-host predictions through the addition of curated prophage sequences to the clustering step and expanded the microbial CRISPR spacer database by 35%. In addition, we have classified 65% of the sequences into prokaryotic or eukaryotic viruses. Third, we have estimated the quality of the UViGs based on their predicted genome completeness. Fourth, we provide visualization in Google Maps of the sample(s) location from which we select virus genomes or genome fragments. And fifth, we enhanced the blast capabilities to query external data (now able to query viral proteins and metagenomic spacers).

Future versions of IMG/VR will boost the data content by adding other predicted viral genomes and genome fragments not easily captured by our current detection pipeline. For example, viral contigs <5 kb are currently filtered out to minimize the rate of false positive predictions. However, this step may also remove many ssDNA viruses (29). Moreover, most NucleoCytoplasmic Large DNA Viruses (NCLDVs) are also filtered out by our current pipeline due to their high number of metabolic genes (30) and the lack of reference genomes when the VPFs were originated. Other virus groups such as virophages are also difficult to identify due to their clear underrepresentation in the databases (31). The development of methods specifically targeting the above viral types, as well as RNA viruses, which can be found in metatranscriptomic samples. We envision IMG/VR as a comprehensive, dynamic, and interactive platform eventually including all types of viruses. Additionally, we plan to improve and expand the host-virus assignments by using a combination of homology-based searches of specific genes and kmer-based machine learning approaches (32,33).

In summary, we anticipate that this improved and updated version of IMG/VR along with the capability of downloading its data content will serve as an essential resource for researchers studying viral ecology and genomics from specific targeted environments to global scales.

## DATA AVAILABILITY

IMG/VR v.2.0 can be accessed directly from its web link: (https://img.jgi.doe.gov/vr) or from the ‘*Data Marts*’ tab in the IMG landing page (https://img.jgi.doe.gov/). As explained under the *‘Downloading’* Results section, IMG/VR v.2.0 is downloadable from the JGI Genome Portals (https://genome.jgi.doe.gov/portal/pages/dynamicOrganismDownload.jsf?organism=IMG_VR) directly or via *Globus Web Application*.

## Supplementary Material

Supplementary Data
