# Supplementary material for: IMG/VR v.2.0: an integrated data management and analysis system for cultivated and environmental viral genomes
Source: Nucleic Acids Res. 2018 Nov 8;47(Database issue):D678–86. doi: 10.1093/nar/gky1127 (PMC6323928; doi:10.1093/nar/gky1127)

SUPPLEMENTARY DATA

Supplementary Figure S1. IMG/VR v.2.0 home page. (A) System analyses features and carts. **(B)** Database content. Direct access to ‘*Viral Datasets*’, ‘*viral Operational Taxonomic Units*’, viral genomes and genome fragments ‘*With Host*’ and predicted viral genomes ‘*Quality*’. **(C)** World interactive Google Map with a geographic location of metagenomic samples. This can be switched to a human body image displaying different body sites when clicking the ‘*Show Human Body Site*’ button. **(D)** Access to UViGs via associated environmental metadata. **(E)** Access to search Capabilities. **(F)** Link to bulk download of database content. Documentation of all the tools and datasets can be found in all the interactive sites in the database.

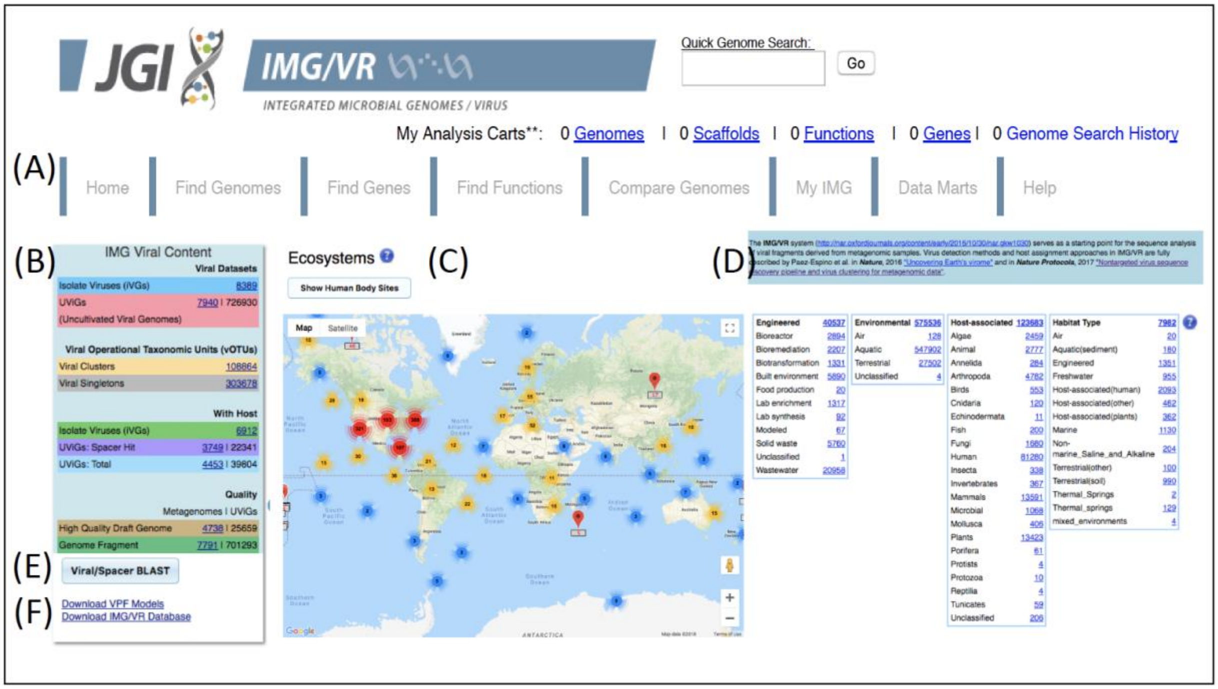

Supplementary Figure S2. Locations to bulk download IMG/VR v.2.0 data. The content of this database can be accessed from (A) the JGI Genome Portals https://genome.jgi.doe.gov/portal/pages/dynamicOrganismDownload.jsf?organism=IMG_VR directly or via *Globus Web Application* or **(B)** the IMG/VR web link: https://img.jgi.doe.gov/vr**.**

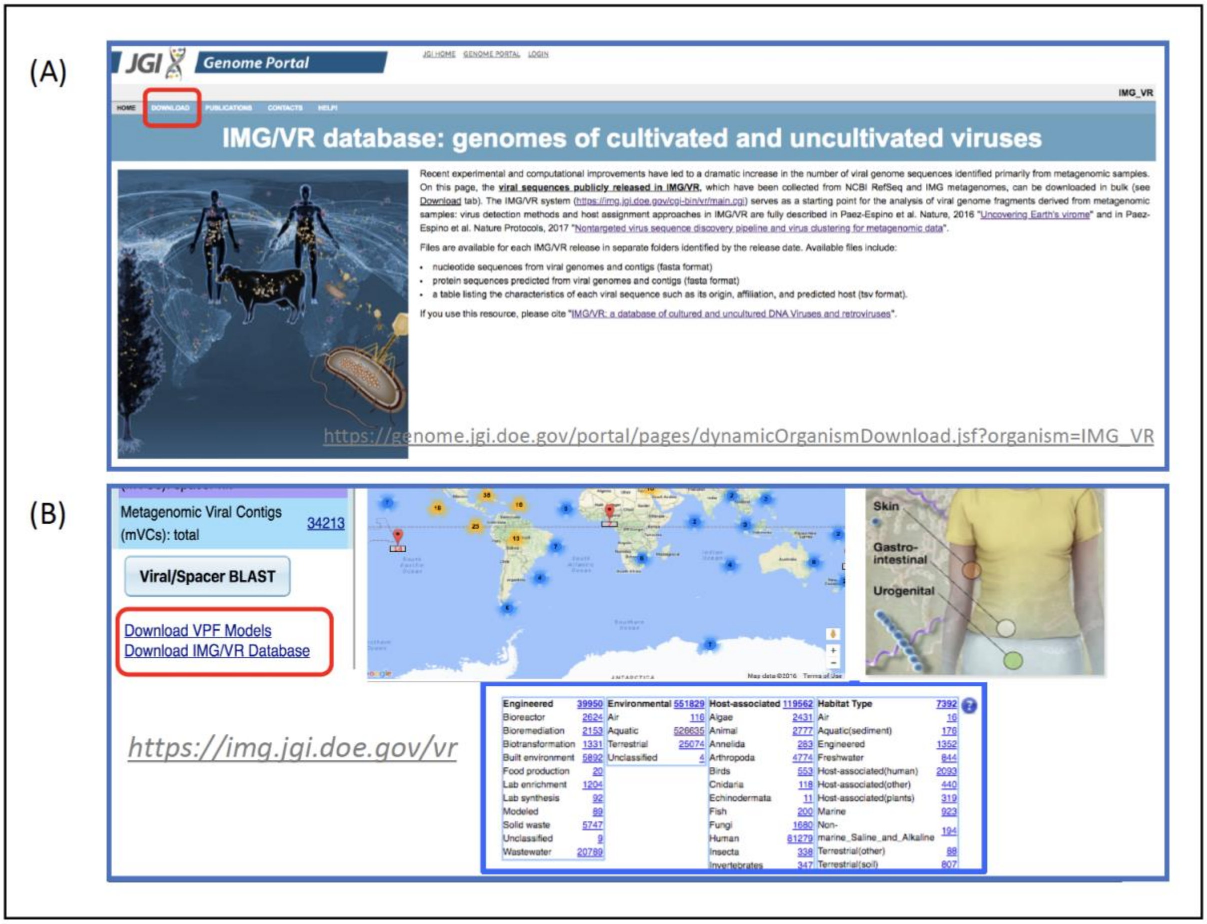

Supplement: Supplementary Data [file gky1127_supplemental_files.pdf]
